# FOXA1 and FOXA2: the regulatory mechanisms and therapeutic implications in cancer

**DOI:** 10.1038/s41420-024-01936-1

**Published:** 2024-04-11

**Authors:** Na Liu, Anran Wang, Mengen Xue, Xiaoren Zhu, Yang Liu, Minbin Chen

**Affiliations:** 1 Department of Radiotherapy and Oncology, Affiliated Kunshan Hospital of Jiangsu University, Kunshan, China; 2 Department of Radiotherapy and Oncology, Gusu School, Nanjing Medical University, The First People’s Hospital of Kunshan, Suzhou, 215300 Jiangsu Province China; 3 College of Life Sciences, University of Chinese Academy of Sciences, Beijing, 100049 China

**Keywords:** Cell death, Cancer, Cancer genetics

## Abstract

FOXA1 (Forkhead Box A1) and FOXA2 (Forkhead Box A2) serve as pioneering transcription factors that build gene expression capacity and play a central role in biological processes, including organogenesis and differentiation, glycolipid metabolism, proliferation, migration and invasion, and drug resistance. Notably, FOXA1 and FOXA2 may exert antagonistic, synergistic, or complementary effects in the aforementioned biological processes. This article focuses on the molecular mechanisms and clinical relevance of FOXA1 and FOXA2 in steroid hormone-induced malignancies and highlights potential strategies for targeting FOXA1 and FOXA2 for cancer therapy. Furthermore, the article describes the prospect of targeting upstream regulators of FOXA1/FOXA2 to regulate its expression for cancer therapy because of the drug untargetability of FOXA1/FOXA2.

## Facts

- FOXA1 and FOXA2 transcription factors in mammals have both overlapping and non-redundant functions.
- FOXA1 and FOXA2 have much crosstalk in steroid-hormone-associated cancers, for example, signaling pathways, epigenetic modifications, and post-translational modifications.
- Both FOXA1 and FOXA2 are involved in cancer resistance, and targeting FOXA1 and FOXA2 can solve the problem of drug resistance.
- Targeting FOXA1/FOXA2 proteins for cancer therapeutic or preventive purposes has become feasible.

## Open questions

- What is the relationship between FOXA1 and FOXA2 in metabolic regulation?
- Will co-targeting FOXA1 and FOXA2 in treating cancer achieve a better therapeutic effect?
- What are the effects of FOXA1 mutations in cancer on FOXA2?
- What are the effects of FOXA2 mutations on cancer development?

## Introduction

**Fig. 1:**
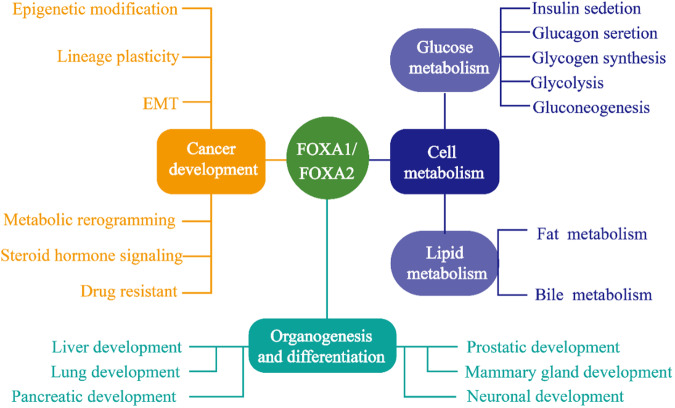
Overview of the functions of FOXA1 and FOXA2 in mammals: please see the text for specific details.

Research into the FOXA (Forkhead box A) family first began in 1990 under the name hepatocyte nuclear factor 3 (HNF-3), of which HNF3α, HNF3β, and HNF3γ are the current members [1]. With the study of its structure and function, HNF3 was renamed FOXA of the forkhead box (FOX) protein family; the three members were named FOXA1, FOXA2, and FOXA3 [2]. So far, researchers have studied FOXA for over 30 years [1]. The members of the FOXA family have evolutionarily conserved DNA-binding domains and are involved in regulating cell growth, differentiation, and embryogenesis [3–5]. FOXA has received increasing attention for its critical roles in several mammalian stages, especially FOXA1 and FOXA2 [4, 6]. Functionally, FOXA1 and FOXA2 are required for the normal development of organs of endodermal origin, such as the liver, pancreas, lungs, and prostate [7, 8]. In addition, FOXA1 and FOXA2 control glucose and lipid metabolism by regulating multiple target genes in the liver, pancreas, and adipose tissue [6, 9]. Mechanistically, the FOXA family of transcription factors maintains enhancer activity and nucleosome localization by opening chromatin structures; thus, they are known as pioneer factors [5, 10, 11]. FOXA1 and FOXA2 play different biological functions in tumor development by regulating the expression of multiple genes, which are closely related to cancer development and chemoresistance [12–15]. This work discusses the roles of FOXA1 and FOXA2 in organ development, glycolipid metabolism, hormone signaling pathway transduction, and cancer drug resistance and their functions as pioneer factor (Fig. 1). The study also highlights that FOXA1 and FOXA2 have the same or opposite roles in several mammalian stages, which provides a theoretical basis for the future treatment of metabolic diseases, neurodegenerative diseases, and cancers by targeting FOXA1 and FOXA2.

## Role of FOXA1 and FOXA2 in organogenesis and differentiation

FOXA family members were initially found in hepatocyte nuclear extracts, and studies have indicated that the initiation of liver development depends on FOXA family members [10, 16]. FOXA1 and FOXA2 activate developmental genes within the foregut endoderm to establish the ability to respond to organ-specific signals and induce the expression of liver-specific genes [16]. Thus, when FOXA1 and FOXA2 are absent, the hepatic gene regulatory network is disrupted, leading to a rapid and substantial reduction in the expression of key hepatic genes, which severely affects liver development and function [10]. FOXA1 and FOXA2 mediate liver development by regulating hepatocyte and cholangiocyte growth [17, 18]. Notably, the deletion of FOXA1 and FOXA2 in mouse embryonic livers results in the proliferation of biliary ductal cells, as FOXA1/2 prevents biliary ductal cells from overproliferation to maintain normal bile duct development [18]. Furthermore, FOXA1 can combine with three other transcription factors, Hnf4a, Prox1, and Hlf, to induce differentiation of liver ductal organoids, which exhibits more hepatocyte-specific features that enhance the transition from biliary ductal cells to hepatocyte lineage [17]. The Notch signaling pathway is crucial in bile duct development, and FOXA1 mRNA levels were found to be significantly lower in Notch2-cKO mice than in control mice, but FOXA2 was normal or elevated [19]. Notch signaling has different effects on FOXA1 and FOXA2 expression [19].

The deletion of FOXA1 and FOXA2 in the mouse pancreatic primordium results in pancreatic hypoplasia [20]. In contrast to normal mice, FOXA1- and FOXA2-deficient mice exhibit hyperglycemia and die shortly after birth [20]. Further studies have shown that FOXA1 and FOXA2 are synergistically involved in regulating Pdx1 (pancreatic and duodenal homeobox 1) expression, affecting pancreatic development [20]. Thus, FOXA1 and FOXA2 are essential transcription factors required for Pdx1 expression and pancreatic growth [20, 21]. Similarly, FOXA1 and FOXA2 regulate signaling and transcriptional programs as are necessary for morphogenesis and cell differentiation during lung formation [22]. In the absence of FOXA1 and FOXA2 in the lung, the branch morphogenesis of the lung would be blocked; however, in the absence of either FOXA1 or FOXA2, the lung morphology does not change in a short time [22, 23]. They may play complementary roles in the superficial regulation of genes required for branch morphogenesis in the lung interstitium during lung morphogenesis [22, 23]. In summary, FOXA1 and FOXA2 have two main aspects during the development of the liver, pancreas, and lung: first, FOXA1 and FOXA2 promote signal-dependent lineage initiation through enhancer initiation; second, FOXA1 and FOXA2 enhance organ cell-type-specific gene expression through lineage-specific recruitment of transcription factors [7].

Studies have detected the expression of FOXA1 and FOXA2 transcription factors in the mouse prostate, which interact with the androgen receptor (AR) to regulate the expression of prostate-specific genes; these transcription factors are crucial to the regulation of prostate morphogenesis and cellular differentiation [13, 24]. In the mammary gland, FOXA1 is required for estrogen receptor alpha (ERα) expression and functional activity, and ERα is essential for mammary gland development and ductal morphogenesis; thus, FOXA1 is necessary for mammary ductal spectrum expansion and morphogenesis [25, 26]. However, the role of FOXA2 in mammary gland development has not yet been investigated.

FOXA1/2 not only regulates organ development but is also a key determinant in neuronal development [27]. Knockout of FOXA1/2 in mouse dopaminergic (mDA) neurons compared with normal mice revealed that FOXA1/2-deficient mice were functionally altered, e.g., severely deficient in food intake, independently of motor control [28, 29]. Mechanistically, FOXA1/2 deletion contributes to the downregulation of tyrosine hydroxylase, the rate-limiting enzyme for dopamine (DA) biosynthesis, leading to reduced DA synthesis and striatal DA delivery [28, 30]. Thus, FOXA1/2 maintains its function by inducing a transcriptional program in DA neurons [28, 30]. FOXA1/2 also mediates neural differentiation. A study overexpressed FOXA1 in P19 cells through adenovirus vectors and found that the sonic hedgehog (Shh) gene associated with early neurogenesis was directly activated by FOXA1 [31]. The dynamic regulation of the Sonic Hedgehog (SHH) signaling pathway by inhibiting FOXA2 expression through retinoic acid (RA) promotes SN (serotonin neuron) differentiation in humans. This study reveals the role of FOXA2 in human SN development and improves its differentiation [32]. Conversely, Notch and Shh signaling mediates the regulation of FOXA1/2 transcription factors through tgf-β1 [33]. Recent studies have shown that LncRNA (NKILA) exacerbates the progression of Alzheimer’s disease by regulating FOXA1-mediated TNFAIP1 transcription [34]. Thus, FOXA1 can regulate neuronal development by itself but also exert biological functions through downstream target genes, which further illustrates the functional diversity and complexity of FOXA.

## FOXA1- and FOXA2-mediated cellular metabolism

### Glucose metabolism

The previous chapter highlights that FOXA1 and FOXA2 synergistically participate in the development of metabolic organs, such as the pancreas and liver, and their functions can be affected by organ hypoplasia [10, 20]. In the pancreas, FOXA1 and FOXA2 alter glucose homeostasis by affecting the function of pancreatic α and β cells to regulate glucagon and insulin secretion [9, 35]. Studies have shown that mice deficient in FOXA1 in the pancreas exhibit hyperglycemia [36, 37]. Mechanistically, FOXA1 deficiency leads to increased expression of uncoupling protein 2, which partially uncouples mitochondrial oxidative phosphorylation, further affecting the subsequent ATP-dependent closure of potassium channels and inward flow of calcium ions, ultimately inhibiting insulin release and leading to hyperglycemia [36, 37]. Alternatively, the absence of FOXA1 may affect the development and maturation of pancreatic β-cells, inhibiting insulin secretion and leading to hyperglycemia [38]. Similarly, FOXA2 controls insulin secretion; however, FOXA1 and FOXA2 have opposing effects on insulin regulation, and the absence of FOXA2 leads to increased insulin secretion and induces hypoglycemia [39]. Specific FOXA2 ablation was shown to cause hyperinsulinic hypoglycemia in both mouse pancreatic tissue and human pancreatic cells [40, 41]. FOXA2 regulates insulin secretion. Conversely, FOXA2 activity is regulated by insulin, which regulates FOXA-2 activity through Akt-mediated phosphorylation and nuclear/cytoplasmic localization [42]. Because of the importance of pancreatic β-cells in diabetes, further understanding of the molecular regulatory pathways of FOXA1 and FOXA2 in pancreatic β-cell development and insulin secretion might provide therapeutic prospects.

FOXA1 and FOXA2 synergistically affect pancreatic α-cell differentiation and control glucagon biosynthesis and secretion [43]. Consistent with reduced glucagon gene expression, glucagon content was decreased in FOXA1- and FOXA2-deficient pancreatic α-cell, and only the combined silencing of FOXA1 and FOXA2 affected glucagon secretion [43]. Furthermore, in mice with endoderm-specific knockout of FOXA2, pancreatic alpha cells did not complete terminal differentiation, resulting in a dramatic reduction in the number of mature alpha cells, triggering hypoglucagonemia and hypoglycemia as well as death [43]. FOXA2 is an important metabolic regulator of glucose metabolism and energy depletion in adipocytes of diet-induced obese mice [44]. It can counteract obesity and diet-induced insulin resistance and maintain glucose homeostasis [44]. FOXA-2 can also activate the expression of insulin-sensitizing genes in adipocytes [44]. Therefore, FOXA-2 is an important activator of glucose metabolism genes.

Hepatic glucose production and consumption is a key centerpiece of systemic glucose homeostasis [45]. The liver regulates glucose production and release through glycogenolysis and gluconeogenesis, so what roles do FOXA1 and FOXA2 play in hepatic glycogenolysis and gluconeogenesis? Studies have shown that gluconeogenesis is compensatory and initiated during fasting to maintain the energy required for human life activities, and FOXA2 plays a central role in the transcription of genes that trigger the initiation of gluconeogenesis [46]. Mechanistically, FOXA2 executes the hepatic gluconeogenesis program by recruiting CREB (cyclic AMP response element binding protein) and glucocorticoid receptor (GR) to their respective target sites in chromatin [46]. Besides, in fasting mice, an up-regulation of ten-eleven-translocation family 3 (TET3) mRNA expression was detected. TET3 was recruited by FOXA2 to HNF4α promoter 2 (P2), leading to promoter demethylation and increased transcription, activation of phosphoenolpyruvate carboxykinase, and the gene encoding glucose-6-phosphatase, and contributing to gluconeogenesis activation, ultimately leading to increased hepatic glucose production (HGP) [47]. TET3 overexpression enhances HGP, and knockdown of TET3 or P2 in the liver improves glucose homeostasis in dietary and genetic mouse models of Type 2 diabetes (T2D). These studies provide potential therapeutic targets for T2D. FOXA1 has been reported to be under-expressed in the kidney tissue of mice with diabetic nephropathy (DN) [48]. FOXA1 overexpression decreased fasting blood glucose and 24 h urinary protein concentration in mice, inhibited the accumulation of mesangial matrix and the proliferation of glomerular basement membrane, and ultimately reduced collagen deposition and interstitial fibrosis in mice kidneys [48]. Mechanistically, FOXA1 binds to the promoter region of SATB1 and inhibits its transcription, leading to the inactivation of the Wnt/β-catenin signaling pathway, thereby inhibiting podocyte apoptosis and DN progression [48].

Adenovirus-mediated FOXA2 transgenic mice exhibited elevated serum bile acid and bilirubin levels and a complete loss of hepatic glycogen stores [49]. This finding shows that FOXA2 plays a negative role in liver glycogen storage [49]. Tian’s group also found that maintaining the expression of HNF6 prevented FOXA2-mediated reduction in the expression of liver glucose transmembrane protein Glut-2 and glycogen levels [50]. In hepatic progenitor cells, FOXA2 inhibits aerobic glycolysis and further inhibits cell proliferation by inhibiting the PI3K/Akt pathway [51]. FOXA2 can prevent glycogen storage and glycolysis to regulate glucose metabolism. In addition, FOXA2 can inhibit PKM2 transcription, affect Wnt/β-catenin protein activity, and block the aerobic glycolysis of thyroid cancer [52]. Therefore, FOXA1 and FOXA2 can indirectly affect the activity of the Wnt/β-catenin signaling pathway and regulate glucose metabolism.

In summary, FOXA1 and FOXA2 control glucose metabolism by regulating multiple target genes in the pancreas, liver, and adipose tissue and are potential therapeutic targets for patients with diabetes. However, when it comes to controlling glucose homeostasis in the pancreas, FOXA1 and FOXA2 work both in opposition and synergy. When the liver and adipocytes control glucose homeostasis, only FOXA2 seems to play a role, and there have been no research reports on FOXA1 so far. Future studies should determine whether FOXA1 is involved in regulating glucose metabolism in the liver and adipocytes.

### Lipid metabolism

Fat metabolism occurs primarily in the liver; metabolic disorders lead to obesity, fatty liver, and other diseases [45, 53]. Studies have shown that by increasing the expression of FOXA2 cDNA in mouse hepatocytes, a large amount of transient steatosis was observed in the liver of mice on the 5th day of life, which was related to fatty acid and triglyceride synthesis, lipid β-oxidation, and amino acid synthesis [45]. FOXA-2 has been shown to inhibit adipocyte differentiation and hinder adipogenesis by activating Preadipocyte factor-1 [44, 54]. FOXA2 deficiency in hepatocytes leads to significant alterations in the expression of key developmental and functional genes in liver progenitor cells and mature hepatocytes, accompanied by massive lipid accumulation, increased bile acid synthesis, and glycerol production but decreased glucose uptake, glycogen storage, and albumin secretion [55]. FOXA2 overexpression in hepatocytes reversed the aforementioned situation and improved hepatocyte function [55]. We speculated that this reversed effect may be because of the different metabolism of the research subjects, and changes in the body’s metabolic rate affect the role of FOXA2. For example, in the fasting (low insulin) state, FOXA2 activates transcriptional programs of lipid metabolism and ketogenesis, whereas, in hyperinsulinemia, FOXA2 is inactivated, thereby promoting hepatic lipid accumulation and insulin resistance [56]. Therefore, FOXA2 may serve as a sensor of metabolism in the body and regulate lipid metabolism in negative feedback. This finding explains the controversial role of FOXA2 in lipid metabolism. Furthermore, FOXA1 reduces lipid accumulation in human hepatocytes, and expression levels are downregulated in non-alcoholic fatty liver disease [57]. In alcoholic liver disease, FOXA1 and FOXA2 appear to act synergistically. Hepatocyte nuclear factor 4, alpha (HNF4α) is an important transcription factor in the liver, which not only regulates the expression of functional genes but also regulates many cellular processes [58]. HNF4a has a decisive effect on hepatocytes and liver functional properties [58]. Notably, the loss of FOXA affects the expression of key liver genes resulting in a breakdown of the liver gene regulatory network [10]. FOXA protein is required for the maintenance of enhancer activity, chromatin accessibility, nucleosome localization, and HNF4α binding. Therefore, the FOXA factor continues to function and maintain the liver gene regulatory network [10]. FOXA1/2 and HNF4a play a coordinated role in the maintenance of hepatocytes and liver function. For example, a specific combination of two transcription factors (Hnf4α + FOXA1, FOXA2, or FOXA3) can induce mouse fibroblasts to directly transform into hepatocytoid cells [59]. Studies have reported that members of the FOXA protein family and Hnf4α sequentially and synergistically bind chromatin to activate liver-specific gene expression [60]. Hippo signaling can affect hepatocyte differentiation by influencing the interaction of HNF4α and FOXA2 with enhancers [61]. Overexpression of miR-122 significantly up-regulates two liver-specific transcription factors FOXA1 and HNF4a, effectively promoting liver differentiation and maturation [62]. In addition, FOXA1/FOXA2 and HNF4α bind to rs953413 on the first intron of ELOVL2, regulating long-chain polyunsaturated fatty acid (LC-PUFAs) metabolism by altering the expression of ELOVL2 [63].

FOXA1- and FOXA2-mediated metabolism not only occurs under physiological conditions but is also widely found in pathological conditions, such as inflammation and cancer [55, 64]. The rapid growth and proliferation of breast cancer cells require more lipid anabolism than normal cells. FOXA1 or FOXA2 triggers lipid metabolism reprogramming by maintaining high expression of endothelial lipase (LIPG) in breast cancer, allowing breast cancer cells to grow and extracellular lipids required for proliferation [64]. The study further showed that the downregulation of LIPG or FOXA in breast cancer resulted in reduced cancer cell proliferation and impaired intracellular lipid synthesis [64]. Therefore, targeting metabolic energy required for survival in breast cancer is also a therapeutic strategy. Thus, FOXA1 and FOXA2 are critical for lipid synthesis and breakdown in breast cancer, suggesting that FOXA1 and FOXA2 are potential therapeutic targets.

FOXA2 is required to maintain normal bile acid homeostasis; thus, studies have identified FOXA2 as a new therapeutic target for liver metabolic diseases [55, 65, 66]. FOXA2 affects the accumulation of bile in the liver by directly or indirectly regulating the expression of genes involved in bile formation and hepatic bile acid transport, including Mrp2, Mrp4, Oatp2, and Mrp3 [65, 66]. FOXA2 deficiency increases cholestatic liver injury in mice [65, 66]. Furthermore, cholestasis in FOXA2-deficient mice in the liver induces inflammatory signaling that activates the mTOR signaling pathway to increase hepatic lipogenesis and obesity in mice [67]. Therefore, maintaining the expression of FOXA2 in cholestatic patients may be a new treatment method for cholestatic liver injury diseases. The latest RNA-sq (RNA sequencing) data demonstrate that after knocking out FOXA1/2, glycolysis, citric acid cycle, and other related genes are down-regulated, and lipid metabolism, pentose phosphate pathway, and ketogenesis are modified, indicating that FOXA1/2 may control the developmental gene regulatory network of the liver, whose knockdown leads to the reprogramming of liver metabolism [68].

FOXA1 and FOXA2 not only play a key role in organ development but also participate in the reprogramming of glucose and lipid metabolism, and they are targets for preventing and treating diabetes, fatty liver, obesity, cholestasis, and cancer. However, our understanding of the metabolism of the FOXA family must be further deepened. Current research on FOXA also has the following shortcomings. First, most studies on FOXA metabolism are animal experiments and lack human clinical trials. Second, the relationship between FOXA1 and FOXA2 in metabolic regulation is unclear. Additional research is required to determine whether the relationship is complementary, synergistic, or antagonistic and is completely dependent on the external conditions and merits. Third, metabolic diseases can be treated by directly knocking out FOXA1 and FOXA2, but this approach also causes the original physiological effects to disappear, leading to side effects such as incomplete organ development and differentiation. We should selectively target the upstream regulatory factors of FOXA1 and FOXA2 in metabolic diseases or downstream regulatory factors to achieve therapeutic effects.

## Role of FOXA1 and FOXA2 in cancers

### Prostate cancer

Globally, prostate cancer (PCa) ranks as the second most common cancer among men worldwide, with the highest incidence in North and South America, Europe, Australia, and the Caribbean [69]. The incidence and mortality of PCa are increasing continuously in China owing to the country’s aging population. According to the World Health Organization’s International Agency for Research on Cancer, there will be more than 115,000 new cases of PCa in China in 2022 [70, 71]. The standard treatment for PCa is surgical or chemical deprivation of androgens called androgen deprivation therapy (ADT), which is initially effective in inducing tumor regression, but in the later stages, most patients become resistant to ADT and progress to castration-resistant prostate cancer (CRPC) and neuroendocrine prostate cancer (NEPC), with a high mortality rate [72–74]. Lineage plasticity is one of the major challenges in the current treatment of PCa [72–74]. The ongoing investigation into the pathogenesis of PCa primarily centers on androgen-transduced oncogenic signaling, whereas FOXA1 and FOXA2 interact with AR to regulate transcriptional programs associated with normal prostate and PCa [13]. Studies have demonstrated that FOXA1 and FOXA2 are not only involved in PCa development but also closely related to their lineage plasticity and drug resistance [75–78]. Therefore, a comprehensive understanding of the mechanism of action of FOXA1 and FOXA2 in PCa can help identify new targets and propose effective combination therapy strategies.

**Fig. 2:**
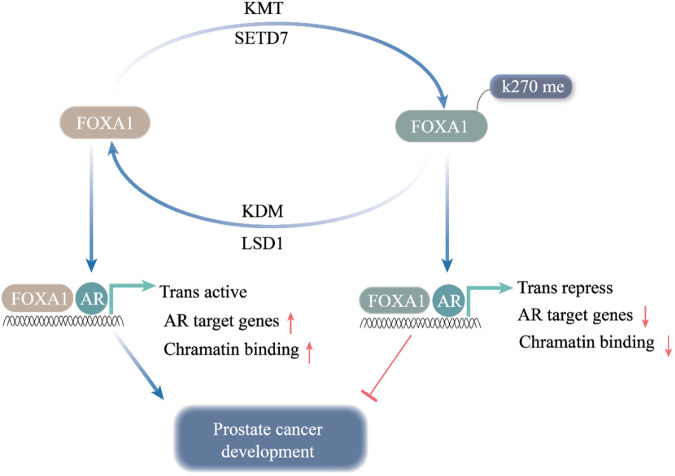
Schematic representation of FOXA1 methylation and demethylation: FOXA1 methylation mediated by KMT (SETD7) results transcription repress; FOXA1 demethylation mediated by KDT (LSD1) promotes chromatin binding, transcriptional activation, and AR downstream signaling. Red arrows in the upward direction indicate facilitation, Red arrows in the down direction indicate inhibition. Blue arrows indicate positive effects. Red perpendicular bars indicate negative effects. Tans-active Transcription active, Trans-repress Transcription repress, K270me methylating lysine at 270 site, KMT Lysine methyltransferase, KDM Lysine demethyltransferase.

Nucleosome localization regulates chromatin open states; DNA located between nucleosomes can bind transcription factors, and their dynamic binding produces different chromatin open states [79]. Specifically, at transcription start sites, FOXA1 replaces nucleosomes and passes through some well-positioned but unstable nucleosomes downstream of the promoter with migration, resulting in nucleosome slip [79]. Thus, it specifically mediates the open state of chromatin structure [79]. FOXA also plays a key role in DNA opening changes because it has a specific recognition function for DNA sequences [79]. FOXA1 induces and opens chromatin conformation and, through direct interaction with AR, creates AR signaling that drives PCa cell growth and survival and is an indicator of PCa progression and prognosis [80]. LSD1 has been reported to act as a transcriptional corepressor and co-activator [81, 82]. LSD1 also acts as a coactivator and a major regulator of AR transcriptional activity [82]. LSD1 interacts with CoREST, binding to and co-activating AR on most androgen-stimulating genes [82]. Furthermore, FOXA1 interacts with the LSD1-CoREST complex and may mediate its initial recruitment of AR regulatory enhancers [82]. LSD1 mediates H3K4me2 demethylation of these androgen-stimulated enhancers to maintain transcriptional inhibition of AR regulatory enhancers [82]. If AR does not enhance H3K4 methylation and FOXA1 binding, LSD1 may mediate demethylation of H3K4me2 at these sites as a negative feedback mechanism to turn off enhancers, thereby preventing abnormal enhancer activation [82]. The transcription profile of LSD1 suggests that LSD1 may stimulate PCa growth through its AR coactivator function and inhibit PCA apoptosis through AR independent mechanism [82]. The transcriptomic analysis results of castration-resistant PCa (CRPC) xenografts sensitive to LSD1 inhibitors show that tumor growth restriction caused by LSD1 inhibition can be attributed to a significant decrease in MYC signal transduction, indicating that MYC is the target of LSD1 [81]. In summary, LSD1 inhibition disrupts the super-enhancer-driven oncogenic transcriptional program in castration-resistant PCa and inhibits PCa growth [81]. Chromatin binding of FOXA1 is facilitated by lysine-specific demethylase 1 (LSD1)-mediated demethylation in PCa [8, 83]. Specifically, LSD1 positively regulates FOXA1 binding to AR and its transcriptional output by demethylating lysine 270 in the wing2 region close to the FOXA1 DNA-binding domain, significantly enhancing PCa growth (Fig. 2) [8, 83]. However, histone-lysine N-methyltransferase SET domain containing 7 (SETD7), the major methyltransferase targeting FOXA1-K270, exerts transcriptional repression in PCa by methylating FOXA1 (Fig. 2) [84]. Furthermore, SETD7 has tumor suppressor activity in PCa cells, and deletion of SETD7 expression is significantly associated with PCa progression and tumor aggressiveness [84]. Therefore, LSD1 and SETD7 are key targets in PCa cells, and targeting LSD1 and SETD7 has been demonstrated to significantly affect PCa growth and aggressiveness (Fig. 2) [8, 83]. FOXA1 expression in PCa not only acts to promote cell growth but also has the effect of inhibiting epithelial-mesenchymal transition (EMT) and preventing cell invasion [85]. Thus, blocking FOXA1 decreases AR transcriptional activity to inhibit PCa cell growth but increases the invasion of PCa cells.

FOXA1 is reported to be a commonly mutated gene in PCa [85]. What effect would its mutation have on the biological behavior of PCa? FOXA1 mutation induces AR reprogramming and transforming growth factor beta (TGF-β) pathway activation to promote EMT-driven cancer metastasis compared with wild type [85]. Comparison of the role of mutant and wild-type FOXA1 in PCa growth showed that patients with FOXA1-mutated PCa had a poorer prognosis; furthermore, almost all FOXA1 mutants resulted in increased cancer cell growth compared with FOXA1 overexpression [76, 86–89]. Mechanistically, FOXA1 mutations promote PCa development by enhancing chromatin binding and inducing AR activation of oncogene expression and EMT signaling pathways [76, 86–89]. In addition, FOXA1 mutations altered its pioneer function and disrupted the normal luminal epithelial differentiation program, further supporting the role of genealogical plasticity in cancer progression [76]. Thus, mutated FOXA1 is an indicator of metastasis and poor prognosis in PCa. However, there have been no studies demonstrating FOXA2 mutations in PCa.

Recent studies have found that PCa cells that express high levels of FOXA1 are capable of driving tumor cell growth, even in the presence of reduced or absent androgen levels, suggesting that tumors with higher levels of FOXA1 may have a growth advantage after androgen deprivation [90]. A plausible explanation may be that through upregulation of FOXA1, which leads to an expansion of the AR chromatin binding range, tumors with high FOXA1 levels may have greater intrinsic AR transcriptional activity and thus be suitable for survival at low levels of androgens [90]. Thus, FOXA1-overexpressing PCa cells are more likely to form CRPC [90]. NEPC—a subtype of CRPC—represents a great challenge for current PCa treatment resistance [91]. The inhibitory role of FOXA1 in neuroendocrine (NE) differentiation and its association with NEPC progression was demonstrated in 2017 [92]. Mechanistically, FOXA1 inhibits its expression by binding to the interleukin 8 (IL-8) promoter, and when FOXA1 expression is decreased or absent, IL-8 up-regulation activates the MAPK/ERK pathway, leading to ERK phosphorylation and enolase 2 (ENO2) expression to promote NE differentiation in PCa cells [92]. Similarly, IL-8 knockdown or ERK inhibition abrogated NE differentiation induced by FOXA1 deficiency [92]. In vitro and in vivo experiments further demonstrated that FOXA1 is deleted or downregulated in NEPC compared with primary PCa and CRPC [92]. Consistent with this result, microRNA-194 (miR-194) was negatively correlated with AR signaling in PCa treated with AR inhibitors, and miR-194 upregulated the expression of the chemokine IL-8 and the EMT transcription factors SNAI2 and ZEB1, which can promote the NE phenotype in PCa, by targeting and inhibiting the expression of the downstream gene FOXA1 [93]. IL-8 upregulation activates the MAPK/ERK pathway, leading to ERK phosphorylation and ENO2 expression, promoting NE differentiation of PCa cells to form NEPC [93]. miR-194 is seen to be a potential target of NEPC [93]. However, FOXA1 is a transcription factor required for NEPC proliferation and NE gene expression, maintaining NEPC proliferation and expression of NE profile-defining genes [77]. The role of FOXA1 in the transition from prostate adenocarcinoma (PRAD) to NEPC seems to contradict the role of maintaining NE gene expression in NEPC. For such results, we make the following speculations. First, FOXA1 mutation may lead to differences in the role of FOXA1 in the NE differentiation of PCa. The authors of the two reports did not indicate whether FOXA1 was mutated or not. FOXA1 is also a common mutational factor in PCa, and mutations in one site can lead to the opposite function. Second, there may be differences in experimental models, with one article using patient-derived xenografts from NEPC and PRAD but the other using PCa cell lines.

FOXA2 also plays a role in the transition of PCa to NEPC [94]. Histone demethylases plant homeodomain finger‐containing protein 8 (PHF8) and FOXA2 are highly expressed in patients with NEPC; PCa is unable to develop into NEPC when the PHF8 gene is absent [94]. PHF8 removes repressive histone marks from the promoter region of the FOXA2 gene by demethylation, contributing to the up-regulation of FOXA2 expression levels, which induces the expression of NEPC developmentally-related genes and drives progression [94]. Thus, the expression levels of PHF8 and FOXA2 can be used as biomarkers for NEPC, and targeting PHF8 or FOXA2 may be a potential therapeutic strategy for NEPC treatment [94, 95]. Subsequent studies have shown that FOXA2 expression is significantly increased after ADT in PCa and drives the transition of PCa to NEPC; notably, FOXA2 knockdown reverses the above transition [75]. Thus, FOXA2 is an important factor driving the transition of PCa to NEPC, as well as a potential therapeutic target for NEPC [75, 94, 95]. Both FOXA1 and FOXA2 play critical roles in the development of NEPC, and whether they are synergistic or antagonistic to each other has not been demonstrated. Based on the available data, we speculated that FOXA1 and FOXA2 play a synergistic role in the transition to NEPC after ADT. Simultaneous blockade of the signaling pathways triggered by FOXA1 and FOXA2 may be the optimal therapeutic option for NEPC.

When PCa is in a hypoxic tumor microenvironment, both FOXA1 and FOXA2 regulate the hypoxic transcriptional program by binding to HIF1A [96, 97]. In CRPC, FOXA1 binds directly to the HIF1A gene enhancer and represses its expression, which, in the absence of FOXA1, leads to increased immunosuppressive macrophage infiltration and cell invasion [96]. This finding is consistent with the conclusion that in CRPC, metastasis is prone to occur with low FOXA1 expression. However, FOXA2 contributes to the upregulation of expression of a subset of HIF1A target genes, including Hes6, Sox9, and Jmjd1a, by binding to HIF1A, stimulating a transcriptional program that ultimately promotes NE prostate tumorigenesis and metastasis [97]. FOXA1 and FOXA2 regulate the hypoxic transcriptional program inversely when PCa is in a hypoxic tumor microenvironment, and which of FOXA1 and FOXA2 tends to regulate HIF1A may depend on the high or low expression of FOXA1 and FOXA2 in the PCa phenotype.

### Breast cancer

According to the latest epidemiological statistics, the incidence of breast cancer in China has reached the top of the female malignant tumors, and the mortality rate ranks fourth in female malignant tumors [71, 98]. Breast cancer is a heterogeneous disease whose subtypes are determined by the presence of estrogen receptor (ER), progesterone receptor (PR), and human epidermal growth factor receptor 2 (HER2); different subtypes are important for clinical management [99].

**Fig. 3:**
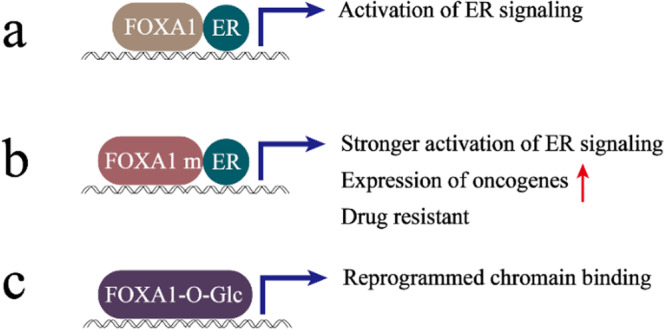
Schematic representation of functional changes of FOXA1 in breast cancer under mutation or epigenetic modification. Please see the text for specific details (Red arrows in the upward direction indicate facilitation. m mutation, O-Glc OGlcNAcylation). **a** FOXA1 activation of ER signaling; **b** effect of FOXA1 missense mutation (Wing2 region) on cancer progression; **c** O-GlcNAcylation of FOXA1 reassembles the binding of FOXA1 to chromatin.

The primary role of FOXA1 is to open tightly coiled chromatin and induce the activation of other transcription factors, including ERα, which is essential for the expression of ERα target genes and the development of estrogen-dependent tissues (Fig. 3a) [100]. In addition, the transcription factor FOXA1 not only affects cell growth but also plays a regulatory role in endocrine therapy sensitivity [101]. Thus, FOXA1 may serve as a key determinant of ER transcriptional programs and endocrine responses in breast cancer [102]. FOXA1 and ER expression are positively correlated in breast cancer, and the vast majority of ER-conducting signals require FOXA1 binding to chromatin for activation, leading to complete suppression of ER transcriptional activity when FOXA1 is absent [14, 103, 104]. As in PCa, mutations of the pioneer transcription factor FOXA1 are common in ER+ breast cancer, and mutation of FOXA1 can alter its function and correlate strongly with endocrine therapy sensitivity [105]. After treatment with an aromatase inhibitor (AI), the progression-free survival of patients with FOXA1 mutations was significantly lower than that of patients with WT FOXA1 [105]. Mechanistically, the FOXA1 missense mutation (Wing2 region) promotes chromatin binding of FOXA1 to ER binding sites and enhances ER-mediated transcriptional signaling under estrogen stimulation (Fig. 3b) [105]. However, in breast cancer, no studies have shown the relationship between FOXA2 and ER, and whether there is a link between the two studies must be further explored in the future.

A growing number of studies have shown that O-linked β-N-acetylglucosamine modification (O-GlcNAcylation) is upregulated in breast cancer cells and is associated with their invasion [106, 107]. In the last few years, the mechanisms of breast cancer O-GlcNAcylation in cancer development have been unraveled. Liu’s group concluded that O-GlcNAcylation of FOXA1 increases the metastatic capacity of breast cancer cells. Mechanistically, O-GlcNAcylation of FOXA1 reassembles the binding of FOXA1 to chromatin and promotes breast cancer metastasis by orchestrating the transcription of a number of metastatic regulators (Fig. 3c) [108]. In addition, the group found that microRNA24 (miR24) inhibited O-GlcNAc transferase to maintain FOXA1 stability and prevent breast cancer cell invasion [109]. Therefore, targeting FOXA1 O-GlcNAacylation is a preventive and therapeutic strategy for metastatic breast cancer.

Regulation of FOXA1 by upstream factors can affect its function, and regulation of FOXA1 expression has been more studied with microRNA [110, 111]. Ectopic microRNA-132 expression significantly inhibited the expression of FOXA1 protein, which also inhibited the proliferation of breast cancer cells, whereas miR-132 knockdown promoted FOXA1 expression in breast cancer cells, which facilitated the proliferation of breast cancer cells [110]. Similarly, microRNA-100 inhibited breast cancer cell proliferation, invasion, and migration by targeting FOXA1 [111]. Overexpression of FOXA1 promoted the proliferation, migration, and invasion of breast cancer cells; thus, the antitumor effect of miR-100 in breast cancer was reversed after FOXA1 overexpression [111]. These results provide new directions for targeted breast cancer therapy.

In addition to its role in ER-positive breast cancer, high levels of FOXA1 improve survival in patients with the basal breast cancer subtype [112]. FOXA1 not only inhibits the molecular phenotype of basal breast cancer cells but also activates the transcription of E-calmodulin and p1Kip51 to block EMT and cell proliferation in basal breast cancer [112–114]. Furthermore, FOXA1 deficiency was able to induce membrane-associated protein 1 (ANXA1) expression, contributing to basal breast cancer metastasis [115]. In vitro and in vivo experiments confirmed that elevated FOXA1 levels inhibited basal cell growth by stimulating the expression of apoptosis-related genes (BIK) and suppressing the expression of cell proliferation-related genes (PCNA, CDC8C, and MCM1) [115]. However, this result was demonstrated in a basal cell line with low expression of Estrogen Receptor-1 (ESR1) [116]. When the cells with high expression of ESR1 significantly reduced the activation of FOXA1 at the BIK promoter, preventing the expression of pro-apoptotic genes and eliminating the inhibitory effect of FOXA1 on cancer development [116]. The role of FOXA1 in basal breast cancer is determined by ESR1 expression [116]. These findings provide a theoretical basis for developing new potential therapeutic strategies for treating refractory basal breast cancer.

FOXA1 plays different roles in different breast cancer subtypes. In ER-positive breast cancer, FOAX1 is an oncogene, whereas in basal breast cancer, the role of FOXA1 is determined by ESR1. Therefore, when targeting FOXA1 for treating breast cancer, we should first distinguish the type of breast cancer, detect the expression of related hormone receptors, and finally decide the treatment plan according to these conditions.

Most research on FOXA2 in breast cancer is related to EMT [117, 118]. In breast cancer, FOXA2 expression prevents breast cancer EMT, and the mechanisms regarding its inhibition are multifaceted [117, 118]. Studies have shown that FOXP2 can inhibit the metastasis of breast cancer cells [119]. The ability of FOXP2 to inhibit EMT in breast cancer is dependent on FOXA2 [119]. Specifically, FOXP2 binds to the promoters of genes associated with breast cancer cells, such as e-cadherin and PHF2, and activates their transcription to inhibit EMT [119]. This transcriptional activity of FOXP2 depends on its interaction with FOXA2 [119]. FOXA2 recruits the transcriptional repressor TLE3 to the ZEB2 promoter and inhibits the expression of the EMT-related transcription factor ZEB2, thus preventing EMT in breast cancer cells. Furthermore, FOXA2 stimulates the promoter of E-cadherin and inhibits EMT formation in tumor cells [117]. In addition to inhibiting downstream EMT-associated transcriptional programs, FOXA2 can be regulated by upstream genes to affect its expression in breast cancer, thereby affecting EMT. Studies have shown that peroxisome gamma coactivator-1β (PGC-1β) is negatively correlated with FOXA2 expression in breast cancer, but PGC-1β binds to FOXA2 to decrease FOXA2 expression in breast cancer and induces EMT [118]. In addition, PGC-1β knockdown and FOXA2 overexpression in breast cancer significantly increased cell proliferation and migration as well as tumor growth [118]. MicroRNA, a common upstream regulator of FOXA2, can affect FOXA2 expression in breast cancer. For example, miR-1290 upregulates ciliary neurotrophic factor (CNTF) expression by decreasing FOXA2 expression in astrocytes, promoting breast cancer cell growth and brain metastasis progression [120]. In triple-negative breast cancer, FOXA2 promotes cell proliferation, maintains cancer stem cells, facilitates the development of triple-negative basal-like tumors, and is associated with recurrence [121]. The results of this study are in complete contrast to previous conclusions that FOXA2 prevents EMT in breast cancer. We speculated that it might be linked to the subtype of breast cancer when there are differences in the expression of ER, PR, and her2, the role of FOXA2 is not the same, and it is possible that the transcriptional program regulation of hormone receptor also influences the direction of cancer, just like FOXA1. Future studies should focus on this aspect and explore these differences to better achieve precision-targeted therapy in tumors.

### Liver cancer

The number of new cases and deaths of cancer in China ranked first in the world. The incidence of liver cancer rose to the fourth place in China, but the mortality rate ranked second [122]. As a common malignant tumor of the digestive tract, liver cancer is mostly diagnosed at an advanced stage and has a poor prognosis [123]. Current immunotherapy and chemotherapy have limited efficacy in treating advanced liver cancer outcomes, and further research is required to find better ways to treat liver cancer [123, 124]. According to statistics, the incidence of liver cancer in men is approximately three times that of women. This difference is mainly because of the difference in sex hormones between men and women, and FOXA1 and FOXA2 are also essential for sexual dimorphism in liver cancer [15, 125, 126]. Starting from the factors that affect the incidence of liver cancer, gaining a comprehensive understanding of the pathogenesis of liver cancer helps explore new treatment options.

Early studies have used the chemical carcinogen diethylnitrosamine (DEN) to induce the formation of liver cancer in mice. Almost all male mice formed obvious liver cancer tumors, whereas only 13% of liver cancers were formed in female mice [125, 127]. Castration in male mice resulted in a decreased incidence of hepatocellular carcinoma (HCC), whereas ovariectomy in female mice resulted in an increased incidence of HCC, suggesting that sex hormones are the major regulators of HCC sexual dimorphism in mice [128]. Further experiments have shown that DEN administration causes a greater increase in serum interleukin-6 (IL-6) concentration in males than in females. When the IL-6 gene was knocked out, it not only reduced the hepatocyte damage caused by the use of DEN in male mice cancer incidence but also eliminated sex differences in liver cancer development in mice [129]. Elevated IL-6 levels contribute to the development of HCC and may be a key factor in the dimorphism of liver cancer [129]. From a mechanistic point of view, the chemical carcinogen DEN induces liver cancer by promoting the production of IL-6 in Kupffer cells (KCs) in a toll-like receptor adapter protein MyD88-dependent manner. Estrogen can inhibit IL-6 secretion by KCs and reduce IL-6 concentration in the circulation of DEN-treated female mice, preventing the formation of liver cancer. Therefore, adding estrogen to male mice can inhibit IL-6 production and reduce the risk of liver cancer in men [129]. This study demonstrates that IL-6 acts as a pro-inflammatory cytokine and is beneficial to the development of liver cancer in a DEN-induced mouse HCC model. Consistent with the pathological conclusion, liver cancer evolves from hepatitis and cirrhosis. Therefore, it seems logical to conclude that IL-6 levels are elevated in patients prone to liver cancer. Recently, in a study by Li’s group, the experiment of using DEN to induce liver cancer in mice was consistent with previous experiments. The formation of liver cancer in female mice was less than one-third that of male mice [15]. This phenomenon can be explained by estrogen preventing the occurrence of liver cancer and androgen promoting the occurrence of liver cancer [15]. However, sexually dimorphic HCC was completely reversed in FOXA1- and FOXA2-deficient mice, with tumor volume becoming larger in FOXA1- and FOXA2-deficient female mice, and conversely, tumor volume was decreased in male mice [15]. The mechanistic prevention of HCC by estrogen and the promotion of HCC by androgens after the deletion of FOXA1 and FOXA2 is not established, and both estrogen signaling that prevents HCC and androgen signaling that promotes HCC are dependent on FOXA1/2 for their existence [15]. Mechanistically, the synergistic regulation of female liver target genes by FOXA1/2 and ERα is enhanced during hepatocarcinogenesis, suggesting that myc is an important target for FOXA2/ERα inhibition in the liver of female mice [15]. In addition, multiple genes involved in cell cycle control and carcinogenesis are dual targets of FOXA/ERα, which are engaged in anticancer-related pathways and prevent hepatocarcinogenesis [15]. The interaction between FOXA1/2 and ERα is lost in FOXA1/2 mutant livers, suggesting that the presence of FOXA1/2 is important for the role of estrogen signaling in the protection of female mice against chemical-induced hepatocarcinogenesis [15]. The incidence of HCC in male mice was decreased with estrogen; therefore, estrogen has a defense-protective effect on the development of HCC [15]. This view conflicts with the pro-carcinogenic role of estrogen in breast cancer, which may be due to the specificity of the cancerous tissue and the internal and external environment in which the cancer is located.

The aforementioned discussion suggests that FOXA1 and FOXA2 are key factors in HCC dimorphism, so what roles do FOXA1 and FOXA2 transcription factors play in the biological behavior of HCC?

As a tumor suppressor, FOXA1 targets PIK3R1 and inhibits the PI3K/Akt signaling pathway, which plays an inhibitory role in the proliferation, migration, and invasion of male HCC patients [130]. Analysis from TCGA data and HCC cases revealed that MCM3AP-AS1, a lncRNA, was overexpressed in HCC and positively correlated with stage and poor prognosis of HCC patients [131]. Mechanistically, MCM3AP-AS1 overexpression inhibited the downstream miR-194-5p expression, which promoted FOXA1 expression and ultimately promoted the growth of HCC [131]. In these two studies, FOXA1 expression is not only inconsistent in HCC but also has different effects on the biological behavior of HCC. We conducted a detailed comparison, and the case samples of the aforementioned two studies were significantly different. First, there was a gender difference in the case samples, with one group being male and the other mixed. Second, the case samples were processed differently, with one group using commercial tissue microarrays to evaluate 90 HCC patients who did not undergo surgery and the other group receiving 80 columns of mixed-gender tissue specimens after hepatectomy. Therefore, we speculated that it might be the variability between specimens that led to the inconsistent results.

FOXA1 is another target gene of miR-3064-5p and miRNA-212, apart from miR-194-5p [132, 133]. miRNA-21, which is downregulated in most HCC tissues, inhibits tumor growth in human HCC by targeting FOXA1 and induces apoptosis in HCC cells [132]. Similarly, miR-3064-5p plays an anti-angiogenic role by inhibiting the FOXA1/CD24/Src pathway, exerting anti-angiogenic effects, and hindering the growth of HCC cells [133]. Therefore, miR-194-5p, miR-3064-5p, and miRNA-212 are potential therapeutic targets for HCC.

FOXA2 is expressed at lower levels in HCC tissues than in paracancerous tissues, and low levels of FOXA2 expression correlate with tumor aggressiveness [134, 135]. An investigation was conducted into the mechanism by which FOXA1 expression was decreased in HCC, which was mainly mediated through long non-coding RNAs (lncRNAs) and micro-RNAs [136, 137]. In HCC, loss of linc00261 reduced FOXA2 expression and finally enhanced the biological behavior of HCC [136]. Conversely, linc00261 inhibited EMT of HCC by promoting FOXA2 expression, thereby inhibiting the migration, invasion, and lung metastasis formation of HCC [136]. Artificial up-regulation of FOXA2 in HCC can inhibit the transcription of matrix metalloproteinase-9 and inhibit the distant metastasis of HCC [138]. In conclusion, FOXA2 acts as a suppressor of biological behavior in HCC. A novel lncRNA named lncRNA-NEF is activated by FOXA2 and frequently downregulated in HCC cell lines and clinical specimens. The ectopic expression of lncrNa-NEF significantly inhibits EMT programming and cell migration [139]. Studies have shown that lncRNA-NEF, a new activator of FOXA2, can interact with β-catenin to increase the binding of GSK3β and β-catenin, thereby inhibiting the Wnt/β-catenin signaling pathway and ultimately activating FOXA2 expression [139]. Therefore, a new positive feedback loop is formed between FOXA2 and lncRNA-NEF to provide new insights into the HCC metastasis process [139]. Low levels of miR-200a were observed in the serum and tissues of HCC patients, miR-200a overexpression in HCC reduced cell proliferation, migration, and invasion, and FOXA2 was identified as a target of miR-200a [137]. The decreased expression of miR-200a in HCC may lead to cell proliferation, migration, and invasion by up-regulating FOXA2 expression, leading to the deterioration of HCC [137]. This study contradicts most studies suggesting FOXA2 as an HCC suppressor gene. We look forward to more studies to explain the dual role of FOXA2 in HCC proliferation, invasion, and metastasis. Besides, it is determined by sex hormones.

### Other cancers

Here, we focus on the role of FOXA1/FOXA2 in cancers affected by steroid hormones, such as PCa, breast cancer, and liver cancer. However, FOXA1/FOXA2 is not only related to the aforementioned three cancer types but also closely related to gastric cancer, colorectal cancer, pancreatic cancer, lung cancer, cervical cancer, endometrial cancer, ovarian cancer, and thyroid cancer [140–146]. Table 1 lists the regulatory mechanisms of FOXA1/FOXA2 in these cancer types.

**Table 1:** Regulatory roles of FOXA1 and FOXA2 in cancer types.

Cancers	FOXA	Upstream factors	Downstream factors	Biological function	References
Gastric cancer	FOXA1 protein reduction	/	/	Inhibits cell proliferation and EMT	[141]
Gastric cancer	FOXA1	/	KRT7	Promotes cell proliferation, migration, and invasion	[171]
Gastric cancer	FOXA1 protein reduction	miR-1290	/	Promotes cell proliferation and metastasis	[172]
Gastric cancer	FOXA2 protein reduction	HDAC3	FTO/m6A/MYC axis	Accelerated cell viability, migration, and invasion	[173]
Gastric cancer	FOXA2 protein reduction	miRNA-187	/	Promotes growth and metastasis of gastric cancer	[174]
Gastric cancer	FOXA2 low expression	/	/	Suppresses gastric tumorigenesis in vitro and in vivo	[175]
Pancreatic cancer	Loss of FOXA1/2	/	/	Induces EMT	[142]
PDAC	FOXA2 low expression	miR-199a	/	Induces an increase in cell proliferation, migration, and invasion.	[176]
Colorectal cancer	/	/	/	Regulates tumor cell growth and predicts prognosis	[177]
Colorectal cancer	FOXA1 was significantly increased	/	YAP	Promotes cell proliferation, migration, and invasion,	[143]
Colorectal cancer	Downregulating FOXA1	miR-93-5p	/	Confer radioresistance	[178, 179]
Lung cancer	Loss of FOXA2 and Cdx2 synergizes with loss of Nkx2-1	/	/	Activates the metastatic program.	[145]
Lung cancer	FOXA1 and FOXA2 expression			FOXA1/2 coordinately regulates the growth and identity of lung cancer	[180]
Lung adenocarcinoma	FOXA1 and FOXA2 expression			FOXA1/2 expression is a lineage-specific vulnerability in NKX2-1-positive LUAD	[12]
NSLCC	Activates FOXA2 expression	LncRNA FTX	/	Inhibit cancer proliferation and metastasis	[181]
Thyroid cancer	FOXA1 protein reduction	miR-132	/	Inhibits cell proliferation, migration, and invasion	[140]
Bladder cancer	Hypermethylation of FOXA1	/	/	Drives squamous differentiation and promotes heterogeneity	[182]
Melanoma	FOXA2 expression was decreased	miR-1246	/	Promotes cell viability and metastasis	[183]
Glioma	Significantly downregulated	/	/	Inhibits invasion and tumorigenesis	[184]
Endometrial cancer	FOXA1 overexpression	/	ERα	Suppresses the progression	[185]
Endometrial cancer	FOXA1 overexpression	/	/	Promotes tumor cell proliferation	[146]
Endometrial cancer	FOXA2	/	/	Suppresses endometrial carcinogenesis and EMT	[186]
Cervical cancer	FOXA1	/	SIX4	Facilitates CC progression and chemoresistance,	[144]
Cervical cancer	FOXA2 low expression	miR-141-3p	/	Promotes cell proliferation, migration, and invasion	[187]
Cervical cancer	FOXA2 ubiquitination	SND1	/	Promotes the migration and invasion capabilities	[188]
Ovarian cancer	Downregulating FOXA2	miR-590-3p	/	Promotes growth and metastasis	[189]

## Targeting FOXA molecules for cancer therapeutics and future research

### FOXA1-targeted combination therapy for PCa treatment

The progression of PCa to CRPC is a major treatment challenge. CRPC tumors become resistant to the antiandrogen enzalutamide through lineage plasticity, and the response to hormones that directly target androgens or ARs, such as enzalutamide, is limited and not durable [147]. Ivermectin—a common anticancer drug—can interact with FOXA1 to reduce AR signaling and chromatin plasticity of G0/G1 cell cycle regulator E2F1 to inhibit cell proliferation [148]. Ivermectin combined with AR signaling inhibition decreases cellular DNA repair capacity, increases intracellular DNA double-strand breaks, and eventually triggers cell death [148]. Thus, inhibition of AR signaling by targeting FOXA1 is a promising therapeutic approach for CRPC [148], which is only a preliminary conclusion in cell lines. Future studies should perform animal models and clinical trials to compare the conventional androgen or AR-targeted therapy with ivermectin in the treatment of PCa and to obtain the most effective treatment regimen for clinical application. Furthermore, poly(ADP-ribose) polymerase-2 (PARP-2) can interact with FOXA1 and promote AR recruitment to the genome-wide prostate-specific enhancer region, which is a key component of AR transcriptional machinery [149]. PARP-2 expression is substantially higher in primary PCa and CRPC than in benign prostate tissues [149]. Li’s group blocked the interaction between PARP-2 and FOXA1 by targeting PARP-2 with the selective PARP-2 inhibitor UPF-1069, thereby attenuating AR-mediated gene expression and inhibiting AR-positive PCa growth [149]. FOXA1 deficiency promotes TGF-β signaling and induces EMT in CRPC, which is blocked by TGF-β receptor I inhibitor LY2157299 [78, 150]. The combination of LY2157299 and enzalutamide in CRPC treatment could exert a synergistic anticancer effect and improve the sensitivity of cancer cells to enzalutamide [78]. In conclusion, targeting FOXA1-mediated oncogenic signaling can significantly inhibit the growth of PCa. Here, we advocate a strategy of targeting FOXA1 combined with endocrine therapy for PCa.

JQ1 is a targeted inhibitor of the bromodomain protein BRD4 and exerts anticancer effects, especially in breast cancer, by inhibiting the epigenetic modification recognized by BRD4 [151, 152]. JQ1 has been shown to interact directly with FOXA1 to reduce AR transcriptional activity and suppress the growth of PCa cells [153]. Concurrently, JQ1 can inhibit FOXA1 to activate multiple invasion pathways, such as BMP signaling and EMT, and induce the upregulation of invasion genes to promote PCa invasion [153]. JQ1 inhibits PCa growth but promotes invasion [153]. We ventured to propose a combination therapy in which JQ1 can be combined with small molecules that inhibit EMT in PCa, such as BMS-345541 -IκB kinase (IKK inhibitor), a potent small molecule compound [154]. First, based on the results of the cell experiment in which the viability and invasion capability of cancer cells were assessed using JQ1 combined with IKK inhibitor compared with the use of JQ1 alone, we speculated that the combination of the two agents not only significantly reduces the proliferation ability of cancer cells but also inhibits the invasion of cancer cells. Second, in vivo experiments or preclinical experiments should be conducted to further verify the results of cell experiments when they are satisfactory.

Furthermore, targeting FOXA1 protein post-translational modification may become one of the approaches for PCa treatment. The interaction between FOXA1 and AR depends on the stability of FOXA1 [155]. Enhancer of zeste homolog 2 (EZH2) methylated FOXA1 at Lysine-295 and enhanced the deubiquitination of FOXA1 by ubiquitin-specific protease 7 (USP7), which maintained the stability of FOXA1 protein and promoted the growth of PCa [155]. Therefore, FOXA1 methylation may be a potential target for PCa treatment [155]. EZH2 enzyme inhibitors could effectively attenuate FOXA1-driven PCa growth alone or in combination with USP7 inhibitors [155]. SETD7 methylates FOXA1 at lysine-270 (K270) to act as a transcriptional repressor in PCa, inhibiting PCa cell viability [8, 84]. These results suggest that FOXA1 methylation leads to opposite signaling in PCa. A reasonable explanation is that various methylation sites of FOXA1 (EZH2 methylates FOXA1 at lysine 295, while SETD7 methylates at lysine 270) lead to opposite functions of the protein. This feature is crucial to PTM and is responsible for the biological functional diversity of proteins [156].

Although numerous studies have shown FOXA1 as a therapeutic target for PCa, FOXA1 is a challenging target due to its extensive regulatory role as a transcription factor. Because of the physiological function of FOXA1 in organ development and metabolism, the safety of FOXA1 targeted therapy in PCa patients is controversial and may seriously affect the quality of life of patients. Therefore, FOXA1-targeted treatment for PCa patients must follow a path that does not endanger the patient’s life and occurs under conditions that reasonably exceed the expected therapeutic exposure; thereby, partial tendencies can be accepted in early clinical development. However, this path advances a known risk to the most expensive stage of development, the clinical stage, and can lead to avoidable clinical toxicity and later failure. Now, scientists are developing innovative strategies to pursue these “unmedicable” targets, including FOXA1.

### FOXA1-targeted combination therapy for breast cancer treatment

In breast cancer, endocrine therapy has become the main treatment for ER-positive breast cancer, but most patients have recurrence and metastasis because of treatment resistance [157, 158]. The regulation of FOXA1-driven ER signaling may be one of the ways to control the progression of breast cancer and improve the drug resistance of breast cancer treatment [159, 160]. Studies have shown that FOXA1 expression is increased in endocrine therapy-resistant breast cancer, and FOXA1 is involved in the formation of drug resistance by regulating downstream gene expression through transcriptional reprogramming [159, 160]. Fu et al. found that FOXA1 gene overexpression triggers oncogenic signatures and proteomic signatures associated with endocrine resistance in an ER-positive endocrine therapy-resistant breast cancer cell line model [159]. IL8 is the most significant gene regulated by FOXA1 transcriptional reprogramming in Endo-R-endocrine-resistant cells, and IL-8 knockdown can inhibit the growth and invasion of tamoxifen-resistant (TAM-R) cells [159]. Therefore, FOXA1 overexpression mediates endocrine resistance by altering ER transcriptome and IL-8 expression in ER-positive breast cancer. However, the expression of FOXA1 and ERα has decreased in TAM-R breast cancer cells, but the activation of transcription factor nuclear factor-κb (NF-κB) and the expression of its target protein IL6 is increased in these cells, contributing to cancer stem cell properties of TAM-R cells [160]. In conclusion, the expression of IL6 and IL8 was significantly increased in TAM-R cells, and FOXA1 affected the endocrine resistance of breast cancer by targeting the downstream factor IL6/8. Therefore, interfering with FOXA1 transcriptional reprogramming in TAM-R breast cancer may be an effective strategy to overcome drug resistance in breast cancer. Endocrine-resistant breast cancer is characterized by a predisposition to distant metastasis, and FOXA1 has been reported to be overexpressed in estrogen receptor-positive (ER+) endocrine-resistant metastatic breast cancer [161]. Mechanistically, upregulated FOXA1 activates the metastatic transcriptional program by driving super-enhancer (SE) reprogramming and recognizes hypoxia-inducible transcription factor (HIF-2a) as a target of FOXA1-induced SE, mediates the expression of metastatic genomes in endocrine-resistant breast cancer cells, and induces proliferation, migration, and invasion of breast cancer cells [161]. From a clinical point of view, the use of HIF-2a antagonists can reduce the proliferation, migration, and invasion of endocrine-resistant breast cancer cells with high FOXA1 expression [161]. In ductal adenocarcinoma of the breast, FOXA1, together with EP300 and RUNX1, enhances E-cadherin promoter activity in metastatic breast cancer cells [114]. FOXA1 overexpression led to increased expression of E-cadherin, which reduced the metastasis potential of breast cancer cells [114]. However, triggering EMT and initiating metastasis induce resistance to endocrine therapy in breast cancer. Here, FOXA1 plays a role in inhibiting cancer cell metastasis and EMT signatures [114]. Many studies have shown that the phosphatidylinositol 3-kinase (PI3K)/Akt/mTOR pathway can mediate resistance to all forms of endocrine therapy [162, 163]. Targeting the PI3K/AKT/mTOR pathway can overcome endogenous therapy resistance [164]. The transcription factor FOXA1 is a key determinant of ER function and endocrine response. What is the connection between the PI3K/AKT/mTOR pathway and FOXA1? Functionally, hormone resistance induced by the PI3K/Akt/mTOR pathway is associated with FOXA1-mediated endocrine therapy resistance [165]. Future studies should explore the relationship between the two mechanisms and drug resistance to endocrine therapy in breast cancer.

FOXA1-mediated transcriptional reprogramming not only leads to endocrine resistance to breast cancer but also leads to bortezomib (BTZ) resistance to breast cancer [166]. By comparing BTZ-resistant breast cancer cells with BTZ-sensitive breast cancer cells, BTZ-resistant breast cancer cells had a high level of O-GlcNAc modification of FOXA1, which altered its function to inhibit the transcription of pro-apoptotic gene Bim and ultimately prevented apoptosis of breast cancer cells [166]. Therefore, the resistance mechanism of BTZ resistance can be speculated to be caused by the O-GlcNAc modification of FOXA1. Further studies showed that treatment of breast cancer with O-GlcNAc inhibitor L01 in combination with BTZ-sensitized BTZ-resistant cells [166]. O-GlcNAcylation of FOXA1 reasseminates FOXA1 binding to chromatin to promote breast cancer metastasis by coordinating the transcription of many pro-cancer cell metastasis regulators [108, 109]. Therefore, targeting O-GlcNA acylation can not only solve the problem of BTZ resistance in breast cancer but also serve as a therapeutic strategy for metastatic breast cancer.

FOXA1 acts as a cis-regulatory element to reprogram ER recruitment, thereby influencing cell growth and response to endocrine therapy [159, 167, 168]. Genome-wide chromatin recruitment, chromatin openness, and transcriptional networks in breast cancer models with frequent FOXA1 mutations found that patients with these mutations had unique chromatin characteristics and did not respond well to AI therapy [105]. Overall, on the one hand, FOXA1 triggers the expression of oncogenic groups associated with endocrine resistance through mediated transcriptional reprogramming, leading to the development of drug resistance. On the other hand, mutations in FOXA1 genes turn on different chromatin regions and activate alternative cistrome and transcriptome involved in regulating the clinical outcome of antiestrogenic therapy [105, 159, 167].

FOXA1 binds to ER and promotes the transcription program of drug-resistance-related genes, which favors cancer progression and drug resistance. Here, we advocate the use of small molecule blockade of FOXA1/ER transcriptional reprogramming in combination with endocrine therapy to address drug resistance in breast cancer. In the future, more drug trials and clinical trials should be carried out to obtain the best combination treatment strategy for breast cancer.

### FOXA2-targeted combination therapy for liver cancer treatment

FOXA2 is not only an important transcription factor for liver development and metabolic homeostasis but also plays a key role in the development and treatment resistance of HCC [15, 55, 134]. A recent study showed that FOXA2 overexpression enhanced the antitumor effect of Lenvatinib. Mechanistically, FOXA2 overexpression enhanced the inhibitory effect of lenvatinib on HCC cells by up-regulating the AMPK-mTOR pathway and promoting autophagy in lenvatinib-treated HCC cells [134]. Therefore, targeting FOXA2 combined with chemotherapy drugs may be an effective treatment for HCC [134]. Starting from the transcriptional and metabolic reprogramming mediated by FOXA1 and FOXA2 in HCC, we expect that more targeted therapies for HCC will be proposed to achieve more precise and effective targets.

## Discussion and perspective

The present article describes in detail the mechanisms and clinical applications of FOXA1 and FOXA2 in organ development and differentiation, metabolic reprogramming, metabolic diseases, and cancer development. Although FOXA1 and FOXA2 are structurally similar, they are not necessarily functionally identical. FOXA proteins promote the binding of nuclear receptors to their respective targets in a context-dependent manner, and their effects largely depend on the biological functions of the respective downstream target genes. FOXA1 and FOXA2 play a synergistic role in organ development, but in some cases, FOXA1 and FOXA2 have opposite effects on glulipid metabolism and cancer development. Notably, the different regulatory functions of FOXA1 and FOXA2 in tumorigenesis are also substantially different in different sexes and various subtypes of cancer, which can act as oncogenes or tumor suppressors. In addition, FOXA is tissue-specific in cancer EMT induction. Finally, the article highlights the role of FOXAs in cancer treatment resistance and proposes strategies for targeting FOXAs in combination with chemotherapy to address cancer resistance.

**Fig. 4:**
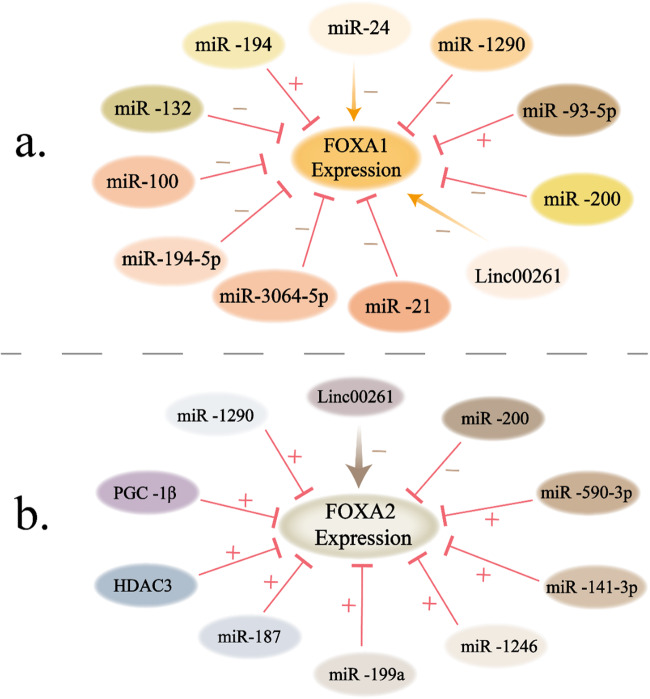
Schematic representation of targeting upstream regulators of FOXA1 and FOXA2 for cancer therapy. Please see the text for specific details (Blue arrows indicate positive effects. Red perpendicular bars indicate negative effects. The minus sign represents an inhibitory effect on cancer growth, and the plus sign represents a promoting effect on cancer growth.) **a** The expression of FOXA1 in cancer is regulated by various factors and has an impact on cancer progression; **b** the expression of FOXA2 in cancer is regulated by various factors and has an impact on cancer progression.

Although many cell and animal experiments have demonstrated that FOXA1/FOXA2 knockout can significantly inhibit cancer progression, FOXAs themselves are undruggable targets for clinical application because of their intricate transcriptional network as transcription factors. Indeed, this finding may also be due to the structural specificity, functional complexity, and diversity of the FOXA family. Based on the aforementioned discussion, we advocate targeting the upstream regulators or downstream target genes of FOXA1/FOXA2 to achieve anticancer effects. In various tumors, FOXA1 and FOXA2 are mostly regulated by microRNA or lincRNA. We can target the upstream regulators that mediate the expression of FOXA1 or FOXA2 to exert anticancer effects (Fig. 4). In addition, targeting FOXA1/FOXA2 epigenetic modification may be an effective approach. At present, it is widely used to degrade protein stability by ubiquitination to counter its function, such as the proteolysis-targeting chimera (PROTAC) method [169, 170]. We hope that more preclinical and clinical trials will be conducted to prove our proposed cancer treatment strategy in the future.
